# Anti-Hcp1 Monoclonal Antibody Is Protective against *Burkholderia pseudomallei* Infection via Recognizing Amino Acids at Asp95-Leu114

**DOI:** 10.3390/pathogens13010043

**Published:** 2023-12-31

**Authors:** Pan Wu, Chenglong Rao, Wenzheng Liu, Ziyuan Zhang, Dongqi Nan, Jiangao Chen, Minyang Wang, Yuan Wen, Jingmin Yan, Juanjuan Yue, Xuhu Mao, Qian Li

**Affiliations:** 1 Department of Clinical Microbiology and Immunology, College of Pharmacy and Laboratory Medicine, Army Medical University (Third Military Medical University), Chongqing 400000, China; panwu183@163.com (P.W.); a15023192726@163.com (W.L.); cjg.yy@163.com (J.C.); lxx911203@163.com (M.W.); wy13102340311@163.com (Y.W.); zijie1011@yeah.net (J.Y.);; 2 State Key Laboratory of Trauma and Chemical Poisoning, Army Medical University (Third Military Medical University), Chongqing 400000, China

**Keywords:** *Burkholderia pseudomallei*, Hcp1, MNGC, monoclonal antibody, T6SS

## Abstract

Melioidosis, a severe tropical illness caused by *Burkholderia pseudomallei*, poses significant treatment challenges due to limited therapeutic options and the absence of effective vaccines. The pathogen’s intrinsic resistance to numerous antibiotics and propensity to induce sepsis during acute infections further complicate management strategies. Thus, exploring alternative methods for prevention and treatment is crucial. Monoclonal antibodies (mAbs) have emerged as a promising strategy for the prevention and treatment of infectious diseases. This study focused on generating three mAbs (13F1, 14G11, and 15D9) targeting hemolysin-coregulated protein 1 (Hcp1), a protein involved in the type VI secretion system cluster 1 (T6SS1) of *B. pseudomallei*. Notably, pretreatment with 13F1 mAb significantly reduced the intracellular survival of *B. pseudomallei* and inhibited the formation of macrophage-derived multinucleated giant cells (MNGCs). This protective effect was also observed in vivo. We identified a sequence of amino acids (Asp95-Leu114) within Hcp1 as the likely binding site for 13F1 mAb. In summary, our findings reveal that 13F1 mAb counteracts infection by targeting Hcp1, offering potential new targets and insights for melioidosis prevention.

## 1. Introduction

Melioidosis, a systemic infectious disease prevalent in Southeast Asia, Australia, and Southern China, is caused by *B. pseudomallei* [1,2]. This disease manifests with symptoms like pulmonary inflammation and persistent cutaneous lesions. In severe cases, it can progress to sepsis, a life-threatening condition with a mortality rate between 20% and 60% [3,4]. The management of melioidosis is challenging due to the intrinsic resistance of *B. pseudomallei* to multiple antibiotics and its ability to survive intracellularly [5,6]. This necessitates prolonged combinational antibiotic treatment, which may contribute to increased antibiotic resistance. Consequently, the development of new preventive and therapeutic strategies is essential.

Monoclonal antibodies (mAbs) have gained prominence as highly specific and reliable tools for treating various diseases, including cancers and autoimmune disorders. Their application in infectious diseases has also progressed significantly [7,8,9]. mAbs can exhibit antibacterial effects by neutralizing toxins, blocking immune evasion mechanisms, preventing bacterial adhesion, and enhancing opsonophagocytic killing (OPK), depending on their target specificity [10,11,12]. For example, mAb therapies targeting key proteins of *Staphylococcus aureus* and *Pseudomonas aeruginosa*, such as ClfA, SraP, SasA, and PcrV, have been well documented [13,14,15,16]. Research has demonstrated the protective role of serum from patients with acute melioidosis in defending THP-1-derived macrophages against *B. pseudomallei* infection, offering insights into antibody-based vaccine development against melioidosis [17]. This study has great clinical value. Although there has been progress in antibody-based treatment of melioidosis, research on mAbs targeting *B. pseudomallei* is still in its infancy, with studies mainly focusing on capsular polysaccharide (CPS) and lipopolysaccharide (LPS) [18,19].

Hemolysin-coregulated protein (Hcp), a critical component in forming the secretion tube of the type VI secretion system cluster 1 (T6SS1), is pivotal in the bacterial infection process. Initially identified in Vibrio cholerae [19], Hcp family proteins have since been recognized for their significance in various bacterial species. For instance, Hcp has been detected in the pulmonary secretions of patients with cystic fibrosis (CF), suggesting a link to the pathogenicity of *P. aeruginosa* [20]. In a rat model of *Escherichia coli* meningitis, the deletion of Hcp resulted in reduced invasion ability of the bacteria into brain tissue [21]. Similarly, in *Burkholderia mallei* and *B. pseudomallei* infections, knockout of the *hcp1* gene led to a significant decrease in intracellular spread and the formation of multinucleated giant cells (MNGCs) [22].

Hcp1 is highly expressed in melioidosis patients and has been shown to induce a cytotoxic immune response in mice infected with *B. pseudomallei*, making it a prominent focus in vaccine research [23,24,25]. Elevated levels of anti-Hcp1 IgG have been found in the serum of melioidosis patients [26], and experiments have demonstrated that 50% of mice immunized with recombinant Hcp1 (rHcp1) were protected from *B. pseudomallei* infection [27]. Furthermore, a staphylococcal membrane vesicle vaccine containing Hcp1 afforded 70% protection in mice against a lethal *B. pseudomallei* challenge [28]. These findings suggest that Hcp1 could be an effective target for the humoral immune response of host against *B. pseudomallei* infection. The potential of Hcp1 has been extensively explored in the diagnosis and prevention of melioidosis [25,28]. However, the protective role of Hcp1 against melioidosis has not been fully revealed, which could provide a novel approach to the clinical prevention and treatment of this disease.

Herein, we present the generation and characterization of three mAbs targeting rHcp1, designated as 13F1, 14G11, and 15D9. Notably, mAb 13F1 demonstrated the ability to reduce the intracellular titer of *B. pseudomallei* in RAW264.7 cells and decrease the formation of macrophage-derived MNGCs. Additionally, 13F1 afforded protection to mice subjected to acute intraperitoneal (i.p.) challenge with *B. pseudomallei*. Our research also suggested that mAb 13F1 bound Hcp1 when *B. pseudomallei* infected host cells and exerted its protective role. Furthermore, we identified that 13F1 bound the amino acid sequence Asp95-Leu114 in Hcp1. These findings could offer promising targets and novel perspectives for the prevention and treatment of melioidosis.

## 2. Materials and Methods

### 2.1. Cell Lines and Bacterial Strains

Murine macrophage cell line RAW264.7 and SP2/0 mouse myeloma cells (Sp2/0-Ag14; CRL-1581) were procured from ATCC (Manassas, VA, USA). These cells were cultured in DMEM medium (Gibco, Carlsbad, CA, USA), supplemented with 10% fetal bovine serum (Gibco, CA, USA), and maintained at 37 °C in a 5% CO_2_ atmosphere. *B. pseudomallei* strain BPC006, a virulent clinical isolate from a melioidosis patient in China [29], was utilized for this study. This bacterium was grown in Luria-Bertani (LB) broth at 37 °C, with shaking at 200 rpm, and on LB agar plates for solid cultures. All experiments involving *B. pseudomallei* were performed in a biosafety level 3 containment laboratory. *E. coli* strains DH5α (CB101) and BL21 (DE3) were acquired from Sangon Biotech (Shanghai, China). The strains used in this study are detailed in Table 1.

### 2.2. Construction of Plasmids and B. pseudomallei Mutant Strains

The *hcp1* gene was targeted for deletion using homologous recombination. Genomic DNA was extracted from the *B. pseudomallei* strain BPC006 using the TIANamp Bacteria DNA Kit (Tiangen, China). The upstream and downstream fragments of *hcp1* were amplified and subsequently cloned into the pK18mobsacB plasmid at the EcoR I-Hind III sites, resulting in the creation of pK18mobsacB-Δ*hcp1*. The pK18mobsacB-Δ*hcp1* plasmid was introduced into *B. pseudomallei* using the S17-1λpir conjugation method. The induction of two rounds of homologous recombination was facilitated by the application of antibiotic and sucrose stress screening. The Δ*hcp1* mutant strain was ultimately confirmed via PCR screening.

We prepared the full-length *hcp1,* which was cloned into the pMLS7 plasmid, a gift from Professor Xiaoqiang Wang of the Tobacco Research Institute, Chinese Academy of Agricultural Sciences, at the Xba I-Hind III sites. This resulted in pMLS7-*hcp1* plasmid. This plasmid was introduced into the Δ*hcp1* strain using the S17-1λpir system. The resulting strain was cultured in LB medium supplemented with 50 μg/mL trimethoprim to ensure the stable presence of the complementary plasmid in the *B. pseudomallei* Δ*hcp1* strain.

DNA sequences encoding different truncated versions of *hcp1*, synthesized by the Beijing Genomics Institute, were cloned into vectors. These variants were inserted into pGEX-6p vectors with an N-terminal GST-tag at the EcoR I-BamH I sites and pET-28a vectors with a 6× N-terminal His-tag at the BamH I-Hind III sites. The specifics of the plasmids and primers used in this study are detailed in Table 1 and Table 2.

### 2.3. Preparation of rHcp1 and Production of mAbs

BL21 (DE3) cells carrying the pET-28a-*hcp1* plasmid were cultured until they reached an OD_600_ of 0.6–0.8. The expression of rHcp1 was induced with 0.5 mM (isopropyl-β-d-thiogalactoside, IPTG) for five hours (Sigma-Aldrich, St. Louis, MO, USA). The cells were lysed by ultrasonication, and rHcp1 was purified using NI-NTA affinity chromatography (GE Healthcare, Chicago, IL, USA) with an AKTA protein purification instrument (GE Healthcare, Chicago, IL, USA). The purity of rHcp1 was confirmed by SDS-PAGE analysis.

BALB/c mice, aged 5–6 weeks, were immunized subcutaneously and intramuscularly with 100 μg of purified rHcp1 protein in phosphate buffer saline (PBS), mixed in a 1:1 ratio with complete Freund adjuvant (Sigma-Aldrich, USA). After three booster immunizations, blood samples were collected to measure serum antibody titers by ELISA (Beyotime, Shanghai, China). Spleen cells from the immunized mice were fused with SP2/0 cells at a ratio of 10:1 and then fused using preheated PEG-2000 (Sigma-Aldrich, MO, USA). The fused cells were cultured on hypoxanthine, aminopterin, thymidine (HAT) semi-solid selective medium for 10 days. Monoclonal hybridoma cells were selected, incubated in 96-well plates for seven days, and then screened using the indirect ELISA method. Approximately 10^7^ positive hybridoma cells were then injected into the abdominal cavity of 8-week-old female BALB/c mice for ascites production. After one week, ascites was collected, centrifuged, and the supernatant was purified using a Protein G antibody purification column (GE Healthcare, Chicago, IL, USA). The purity of the antibodies was monitored by SDS-PAGE.

### 2.4. Rate of Association/Dissociation

Kinetic interactions between full-length Hcp1 and anti-Hcp1 mAbs were measured by Surface Plasmon Resonance (SPR) on BiacoreT200. Hcp1 was diluted to 2 μg/mL in immobilization buffer and injected into a sample channel (Fc2) at a flow rate of 10 μL/min. Immobilization levels were typically kept at 1900 resonance unit (RU) and the reference channel (Fc1) did not need a ligand immobilization step. 13F1, 15D9, and 14G11 were diluted to 8 concentrations (400, 200, 100, 50, 25, 12.5, 625, and 0 nM) and injected to channel Fc1–Fc2 at a flow rate of 30 μL/min for an association phase of 120 s, followed by 300 s dissociation. The binding phase was used to determine the association constant (ka) between mAbs and Hcp1. The dissociation phase (kd) was measured using the rate of decline in RU on introduction of free buffer at the end of mAbs injections. Data were fitted by Biacore T200 Evaluation Software and the dissociation constant (KD) of the complexes was determined as the ratio kd/ka.

### 2.5. Quantitative RT-PCR (qRT-PCR) and Western Blotting

Utilizing the PrimeScript RT-PCR kit (Takara, Beijing, China) and SYBR Green Realtime PCR Master Mix (Thermo Scientific, Waltham, MA, USA), qRT-PCR analyses for the mRNA of *hcp1* and *vgrG* were performed. The reaction conditions were 95 °C for 1 min, followed by 45 cycles of 95 °C for 15 s, 60 °C for 2 min, and 72 °C for 1.5 min for elongation. The mRNA level of 16S rRNA was used as an internal control to normalize the data.

Total proteins were separated using 10% SDS-PAGE and transferred onto PVDF membranes (Millipore, Burlington, MA, USA). Membranes were blocked with triethanolamine-buffered saline (TBS) solution containing 5% skim milk for 2 h at room temperature (RT) and incubated with primary antibodies overnight at 4 °C. Following washing with TBST (TBS with 0.1% *v*/*v* Tween20), membranes were incubated with HRP-conjugated antibodies (Beyotime, Shanghai, China) or goat anti-rabbit fluorescence-conjugated antibodies (Sigma, St. Louis, MO, USA) for 2 h at RT. Detection was performed using the ChemiDocTMTouch Imaging System (Bio-rad, Hercules, CA, USA) and iBright 1500 imager (Thermo Fisher Scientific, Waltham, MA, USA).

### 2.6. Mouse Survival and Organ Burden

Specified pathogen-free (SPF) female BALB/c mice, aged 6–8 weeks (Vital River Laboratory Animal Technology Co., Beijing, China), were adapted to the environment for a week. They were then passively immunized by i.p. injection of 20 mg/kg nonprotective mouse IgG (NP), 13F1, 14G11, or 15D9. Twenty-four hours after immunization, mice were challenged with *B. pseudomallei* (4 × 10^5^ CFU) via i.p. injection. Survival was monitored for five days (*n* = 12). Forty-eight hours post-infection, livers and spleens were harvested (*n* = 6), lysed with 0.1% Triton-X 100 (AMRESCO), and plated on LB agar plates. After 36 h of incubation at 37 °C, colonies were counted to assess bacterial load. All animal experiments were conducted in a BSL-3 biohazard animal room under barrier conditions and approved by the Laboratory Animal Welfare and Ethics Committee of the Army Medical University (AMUWEC20230034).

### 2.7. Cell Infection and Experiment on Bacterial Intracellular Survival

RAW264.7 cells were pretreated with either 50 ng/mL of mAb or NP for two hours. The cells were then infected with *B. pseudomallei* strain BPC006 (cultured to an OD_600_ of 0.6–0.8) at a multiplicity of infection (MOI) of 10:1. After two hours of infection, 250 μg/mL kanamycin was added to the culture to eliminate extracellular bacteria. Two hours after kanamycin treatment, the cells were incubated in a regular culture medium for an additional five hours. Cells were permeabilized with 0.1% *v*/*v* TritonX-100 for five minutes at RT. The resulting supernatants were collected, serially diluted, and plated on a solid LB medium with four replicates for each dilution. Colony-forming units (CFUs) were quantified after 36 h of incubation at 37 °C by counting individual colonies.

### 2.8. Giemsa Stain

RAW264.7 cells were stained using a Giemsa staining kit (BBI Life Sciences Corporation, Shanghai, China). Briefly, approximately 1000 cells from each group were counted under a microscope at ×400 magnification. Cells containing more than three nuclei were classified as MNGCs. The fusion index was calculated as the ratio of the total number of nuclei within MNGCs to the total number of cells. The average MNGC size was determined by dividing the total number of nuclei in MNGCs by the number of MNGCs.

### 2.9. Immunofluorescence Assay

Infected cells were fixed using a solution of 4% paraformaldehyde and 0.3% *v*/*v* TritonX-100 for 10 min. Cells were stained using a rabbit anti-*B. pseudomallei* antibody or mouse anti-Hcp1 antibody 13F1 prepared in the lab, conjugated with Alexa Fluor 488. The antibody was diluted 1:500 and incubated with the cells for one hour. The cytomembrane was stained using rabbit anti-Na^+^/K^+^-ATPase (Santa, Shanghai, China), conjugated with Alexa Fluor 595. The cytoskeleton was stained with Alexa Fluor 594 phalloidin, and nuclei were stained with 4,6-diamidino-2-phenylindole (DAPI), both from Beyotime, China [32]. Following incubation, the cells were washed twice with PBS and examined using confocal microscopy.

### 2.10. Statistical Analysis

Each experiment was conducted three times independently. Results are shown as the mean ± standard deviation (SD) from at least three independent experiments. The Student’s *t*-test or two-way ANOVA was employed for data analysis. Data analyses and graph visualizations were performed using GraphPad Prism software. Statistical significance was set at *p* < 0.05. Asterisks (* *p* < 0.05, ** *p* < 0.01) indicate statistically significant differences, while “ns” denotes no significant difference.

## 3. Results

### 3.1. Anti-rHcp1 mAbs Reduce the Intracellular Survival of B. pseudomallei and Exhibit Protective Capacity In Vivo

Hcp1 is identified as a primary virulence factor in the T6SS1 of *B. pseudomallei* [28,33], playing a pivotal role in MNGC formation (Supplementary Figure S1). To investigate the potential of Hcp1 as a target for mAb therapy, BALB/c mice were given three booster immunizations with rHcp1, produced in *E. coli* (Supplementary Figure S2A,B). This led to the purification of three mAbs from mouse ascites, characterized by high purity and titers (Supplementary Figure S3A,B). The specificity of these mAbs was validated through immunoblotting (Supplementary Figure S3C). For comparison, recombinant BipD (rBipD), a T3SS needle tip protein with a His-tag, was also purified in our laboratory.

**Figure 1:**
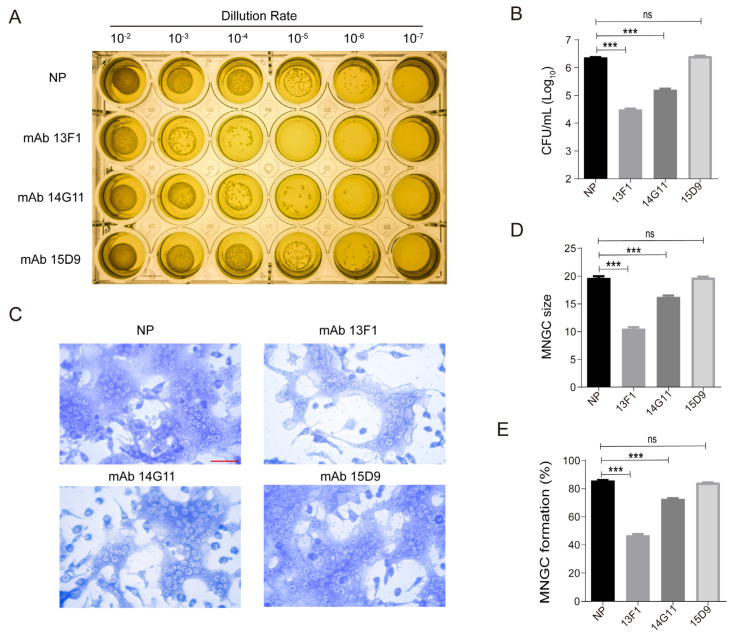
The macrophage–derived MNGC formation and intracellular survival of *B. pseudomallei* in RAW264.7 cells are reduced by mAbs 13F1 and 14G11. (**A**,**B**) 13F1 and 14G11 decreased the intracellular survival of *B. pseudomallei* in RAW264.7 cells. (**C**) Giemsa stain images show that MNGC formation was blocked by 13F1 and 14G11. (**D**,**E**) Quantification of average MNGC size and MNGC formation proportion in (**C**) from three independent experiments, counting approximately 1000 cells each time. NP is nonprotective mouse IgG. Data are the mean ± standard deviation of the means from three independent experiments. *** *p* < 0.001, ns, no significance, control vs. treatment. Scale bar, 50 µm.

The protective effectiveness of these mAbs was evaluated through experiments on intracellular survival and MNGC formation. Figure 1A reveals that mAbs 13F1 and 14G11 diminished the intracellular survival of *B. pseudomallei* in RAW264.7 cells. Remarkably, 13F1 notably reduced intracellular bacterial growth by over 10 times compared to the NP group, as shown in Figure 1B. Given the essential role of Hcp1 in T6SS1 assembly and MNGC formation, as indicated in references [34,35], it was hypothesized that the decrease in intracellular survival could be associated with disrupted MNGC formation. This was confirmed by assessing the influence of mAbs on MNGC formation in RAW264.7 cells using Giemsa staining, as depicted in Figure 1C. Both 13F1 and 14G11 mAbs notably reduced the proportion and size of MNGC, particularly 13F1, which was most effective (Figure 1D,E). 

### 3.2. Anti–rHcp1 mAbs Exhibit Protective Capacity In Vivo

**Figure 2:**
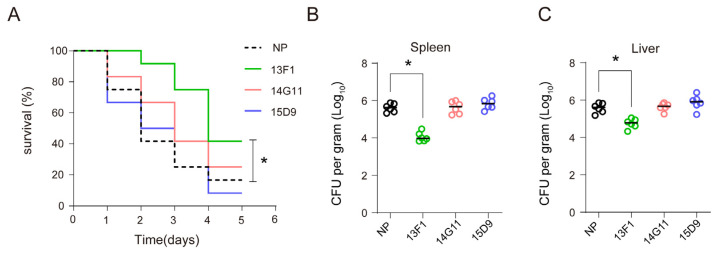
Protective effect of 13F1 on *B. pseudomallei*–induced mouse infection models. (**A**) Survival ratio of *B. pseudomallei* infected mice in the presence of mAb treatment. BALB/c mice (*n* = 12) were injected i.p. with 20 mg/kg of 13F1, 14G11, 15D9, or NP 24 h prior to i.p. challenge with *B. pseudomallei* (4 × 10^5^ CFU), and survival was monitored for five days. Statistical analysis was performed with a log-rank test (Mantel Cox test). (**B**,**C**) Bacteria were enumerated in spleens or livers 48 h after infection (*n* = 6). Horizontal lines represent geometric mean numbers of CFU. Statistical analyses were conducted with a Mann–Whitney U test. Data are representative of three independent experiments. * *p* < 0.05, ns, no significance, control vs. treatment.

Furthermore, to determine the effect of these anti–Hcp1 antibodies on the virulence and intracellular survival of *B. pseudomallei* in vivo, BALB/c mice were infected with the bacteria via i.p. injection and treated with mAbs. The survival of the mice and the bacterial load in their spleens and livers were monitored. Compared to the NP group, treatment with 13F1 showed a protective effect in mice (Figure 2A) and reduced CFU counts in spleens and livers (Figure 2B,C). However, mAbs 14G11 and 15D9 did not exhibit similar survival benefits or reduction in bacterial load in organs. In summary, these findings indicate that mAb 13F1 effectively reduced the intracellular survival of *B. pseudomallei* and offered protective capabilities in vivo.

### 3.3. The Epitope Recognized by 13F1 Is Specifically Located Hcp1 Asp95-Leu114

**Figure 3:**
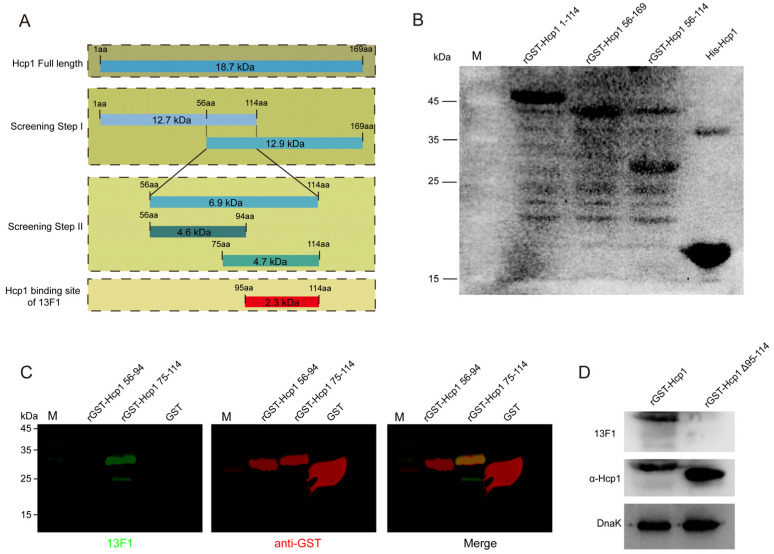
13F1 mAb specifically recognizes Hcp1 Asp95–Leu114. (**A**) Illustration of pGEX–6p constructs with Hcp1 mutant proteins and N–terminal GST fusions indicated. (**B**) Western blotting images show that 13F1 combined Hcp1 56–114. (**C**) Fluorescent Western blotting reveals that 13F1 bound to Hcp1 Asp95–Leu114. (**D**) Immunoblots using monoclonal 13F1 or rabbit polyclonal antibodies (α–Hcp1) to detect rGST–Hcp1 or its variant lacking residues 95 to 114 (rGST–Hcp1 Δ95–114). α–Hcp1 was prepared by our laboratory.

The next phase of our research aimed to ascertain the subclass and affinity of the monoclonal antibody 13F1 and identify its antigen-binding region. ELISA results, presented in Supplementary Figure S4A, identified 13F1 as belonging to the IgG1 subclass. The dissociation constant KD for the binding of MAb 13F1 to Hcp1 was calculated at 8.22 nM (Supplementary Figure S4B). To determine the specific epitope recognized by 13F1, a two-step truncation approach was employed, as detailed in Figure 3A. Initially, we expressed three variants of rGST–Hcp1 (52 kDa): rGST–rHcp1 1–114 (46 kDa), rGST–rHcp1 56–169 (40 kDa), and rGST–rHcp1 56–114 (31 kDa). The binding of 13F1 to all three recombinant proteins suggested its recognition region was within the Hcp1 fragment 56–114, as shown in Figure 3B.

Further refining our analysis, three more recombinant proteins were constructed: rGST–rHcp1 56–94 (31 kDa), rGST–rHcp1 75–114 (35 kDa), and rGST–Hcp1 Δ95–114 (50 kDa). Intriguingly, while 13F1 showed reactivity with rGST–Hcp1 75–114, it did not react with rGST–Hcp1 56–94, as evidenced in Figure 3C. Furthermore, we found that 13F1 did not bind to rGST–Hcp1 Δ95–114 (Figure 3D). These results indicated that the epitope recognized by 13F1 was specifically situated within the Hcp1 region from Asp95 to Leu114. Located between β–sheets 4 and 5 of the Hcp1 protein, these residues, as revealed by structural analysis in Supplementary Figure S5, might play a vital role in the assembly of the T6SS tubular system.

### 3.4. 13F1 Plays Its Protective Role by Binding Hcp1

To understand the process by which mAb 13F1 inhibits the formation of macrophage–derived MNGCs and reduces the intracellular survival of *B. pseudomallei* in RAW264.7 cells, we constructed a *hcp1* deletion *B. pseudomallei* strain (Δ*hcp1*) and a *hcp1* complemented *B. pseudomallei* strain (Δ*hcp1*: *hcp1*). And RAW264.7 cells were infected with three strains of *B. pseudomallei*: the wild type, Δ*hcp1*, and Δ*hcp1*: *hcp1*, in the presence of either mAb 13F1 or mouse IgG.

**Figure 4:**
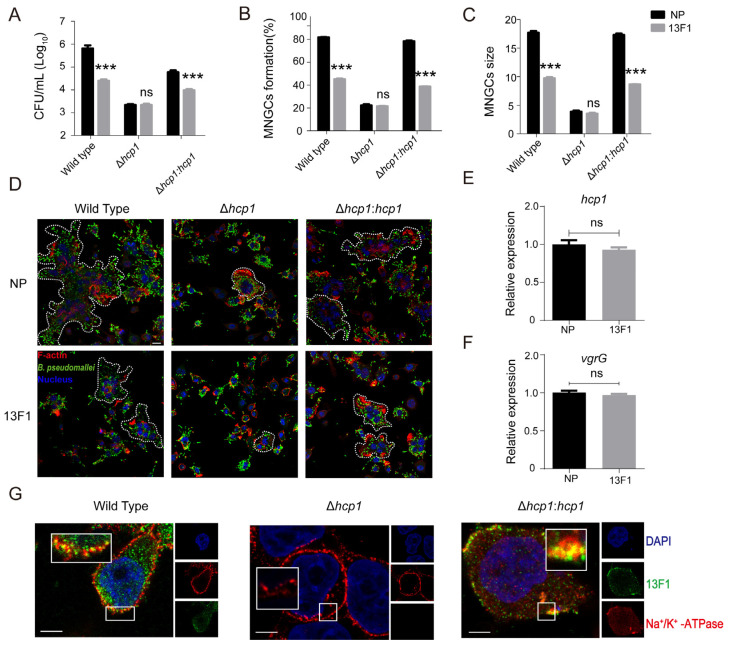
13F1 reduced the macrophage–derived MNGC formation and intracellular survival of *B. pseudomallei* by combining Hcp1. (**A**) After pretreatment with 13F1 or NP, RAW26.7 cells were infected with wild type, Δ*hcp1*, and Δ*hcp1*: *hcp1* for 10 h. The intracellular survival of *B. pseudomallei* in each group was then measured and counted. (**B**,**C**) RAW264.7 cells were infected with *B. pseudomallei* in the presence or absence of 13F1 for 10 h; the proportion and size of MNGC formation were counted. (**D**) Example of immunofluorescence images from the analysis in (**B**,**C**). Scale bar, 20 µm. (**E**,**F**) Transcription analysis of the T6SS1 components *vgrG* and *hcp1*. (**G**) Host cells were infected with *B. pseudomallei* wild type, Δ*hcp1*, and Δ*hcp1*: *hcp1* (from left to right). Boxes indicate the regions of interest. Mammalian cell nuclei were stained using DAPI (blue) and the cytomembrane and Hcp1 was stained with anti–Na^+^/K^+^–ATPase (red) and 13F1 (green), respectively. Scale bar, 2.5 µm. Data are the mean ± standard deviation of the means from three independent experiments. *** *p* < 0.001, ns, no significance, control vs. treatment.

Consistent with prior observations, treatment with 13F1 led to a significant reduction in the number and size of MNGCs, as demonstrated in Figure 4D. Obviously, the 13F1 treatment showed no additional significant effect (reduction in MNGC formation) after the deletion of *hcp1* (Figure 4B,C). Furthermore, 13F1 was still unable to reduce the intracellular survival of *B. pseudomallei* in the Δ*hcp1* group (Figure 4A). Additional experiments revealed that the transcription of key T6SS1 components, such as *vgrG* (encoding valine–glycine repeat protein G, a needle component of *B. pseudomallei* T6SS1) and *hcp1*, remained unaffected by 13F1 treatment (Figure 4E,F). We found that Hcp1 stained with 13F1 was co–localized with the cytomembrane marker Na^+^/K^+^–ATPase when *B. pseudomallei* wild type and Δ*hcp1*: *hcp1* infected host cells, while the co–localization was eliminated when *hcp1* was knockout (Figure 4G). This result was similar to the findings of Gan et al. [33]. Simultaneously, Figure 4G suggested that 13F1 might bind to Hcp1 when *B. pseudomallei* infected host cells. In short, these findings indicate that 13F1 reduced the macrophage–derived MNGC formation and intracellular survival of *B. pseudomallei* by binding Hcp1.

## 4. Discussion

In this section, the focus is on the intricate role of T6SS1 in interactions with mammalian hosts, mediating cytotoxicity, intracellular dissemination, and replication by delivering effectors into host cells [33]. Hcp1, a pivotal component of T6SS1, is integral to the pathogenesis of *B. pseudomallei* [36], necessitating further exploration of its role in infection. The study presented new insights into the protective application of anti–Hcp1 mAb and identified that 13F1 recognized a 20–amino–acid segment in Hcp1. This discovery could pave the way for novel preventive and therapeutic strategies against melioidosis.

The T6SS, constituted of 13 core genes, resembles a membrane–spanning syringe with a structure like a bacteriophage tail. It is increasingly recognized as vital in promoting intracellular replication and cytotoxicity in host cells [36,37]. Hcp, essential for assembling a functional T6SS, facilitates the injection of T6SS substrates by chaperoning the substrates [38]. Hcp1, a member of the Hcp protein family, is crucial in the infection processes of various bacteria, such as *E. coli* and *Aeromonas hydrophila* [39,40]. The structure of Hcp1, comprising nine β–sheets and one α–helix forming a hexameric ring, is fundamental to its function. While the functional regions of Hcp1 have been mapped, studies focusing on its extended loop are limited [34,41].

For instance, research [41] on the Hcp1 hexameric ring of *Campylobacter jejuni* highlighted its significance in cytotoxicity towards HepG2 cells, with an Arg–to–Ala mutation at position 30 in the extended loop reducing this cytotoxicity. Conversely, *B. pseudomallei* Hcp1 have a unique extended loop (Asp40–Arg56) compared to other Hcp homologs, with mutations in key loop residues (Gln46 and Glu47) affecting MNGC formation in host cells infected with *B. pseudomallei*. Distinct from the extended loop examined in the previous studies, 13F1 bound to residues Asp95–Leu114 of Hcp1, which forms a β–sheet. Our results revealed that 13F1 mAb specifically recognizes Hcp1 Asp95–Leu114. However, we are still not sure whether 13F1 plays an antibacterial effect by binding the Hcp1 Asp95–Leu114. Meanwhile, whether Asp95–Leu114 plays a vital role in the assembly of the T6SS1 tubular system remains to be further studied, which is essential to understand the potential mechanisms of preventive applications of targeting Hcp1.

The growing body of evidence underscores the effectiveness of mAbs in treating infectious diseases [42,43]. The protective mechanisms of different mAbs are closely linked to their specific targets. For instance, the anti–SraP mAb, targeting a serine–rich repeat protein from *S. aureus*, prevents bacterial adhesion and invasion into host cells by blocking its binding to sialylated receptors [14]. Similarly, mAbs against PcrV and LcrV, the type III secretion needles of *P. aeruginosa* and *Y. pestis*, respectively, thwart bacterial immune evasion [44,45]. In the context of *B. pseudomallei* infection, MNGCs provide a conducive environment for bacterial growth and spread, a process distinct from that in *Mycobacterium tuberculosis* infection [46]. *B. pseudomallei* T6SS1 is known to play a critical role in MNGC formation in infected host cells. Gan et al. [34] revealed that Hcp1 may localize to the surface of *B.–pseudomallei*–infected cells by binding to membrane receptors. By staining with 13F1, we also found that Hcp1 was localized in the host cell membrane and co–localized with Na^+^/K^+^–ATPase. This study contributes to this body of knowledge by demonstrating that mAb 13F1 inhibits MNGC formation and pathogenic activity post *B. pseudomallei* infection. It is postulated that 13F1 might hinder the interaction between Hcp1 and its membrane receptors in infected host cells, thereby impairing T6SS1–induced pathogenesis in a manner dependent on binding Hcp1.

In order to explore whether Hcp1 could be used as a therapeutic and protective target for acute infection of *B. pseudomallei*, we used an acute infection model in BALB/c mice. Similar to these previous studies [47,48], we found that 13F1 reduced the number of CFU in the spleens and livers of infected mice and doubled the survival rate of the mice in a five–day observation. Next, in order to comprehensively evaluate the protective effect of 13F1, other appropriate infection models will be used.

In summary, the study confirmed mAb 13F1 not only reduced MNGC formation and intracellular bacterial growth but also demonstrated protective capacity in vivo and then further discovered that 13F1 mAb specifically recognized Hcp1 Asp95–Leu114. Finally, our results showed that mAb 13F1 combined Hcp1 when *B. pseudomallei* infected host cells and exerted its protective role. This research lays the groundwork for developing immunotherapeutic and immunoprotective agents, particularly by enhancing the binding affinity of the 13F1 mAb. Future research should aim to unravel the mechanisms underlying MNGC formation, offering potential strategies to combat *B. pseudomallei* infection more effectively.

## Figures and Tables

**Table 1:** List of plasmids and bacterial strains used in this study.

Strain or Plasmid	Relevant Characteristic	Source of Reference
Plasmids		
pMLS7	Broad-host-range vector; Tp^r^; S7 ribosomal protein promoter from *Burkholderia* sp. strain LB400	Lefebre, M.D. et al., 2002 [30]
pMLS7-*hcp1*	pMLS7 containing a 510-bp full-length of *B. pseudomallei hcp1* gene	This study
pET-28a-*hcp1*	pET-28a containing a510-bp full-length of *B. pseudomallei hcp1* gene	This study
pET-28a-*hcp1_283-342-null_*	pET-28a containing a 450-bp length of *B. pseudomallei hcp1* gene	This study
pK18mobsacB	Conjugative, suicide vector	Wong, J. et al., 2015 [31]
pK18mobsacB-Δ*hcp1*	pK18mobsacB containing 802-bp fragment upstream and 550-bp fragment downstream of ORF BPC006_RS26143	This study
Strains		
*E. coli*		
DH5α	Cloning host	Sangon Biotech
DH5αBL21	Cloning host	Sangon Biotech
DH5αS17-λpir	Donor strain for conjugation	Wong, J. et al., 2015 [31]
*B. pseudomallei*		
BPC006	*B. pseudomallei* wild-type strain	Fang, Y. et al., 2012 [29]
Δ*hcp1*	BPC006 Δ*hcp1*, codon 1-510 of BPC006_RS26143 ORF were deleted	This study
Δ*hcp1*:*hcp1*	Δ*hcp1* with plasmid pMLS7-*hcp1*	This study

Tp^r^, trimethoprim resistance.

**Table 2:** List of PCR primers used in this study.

Primer Name	Sequence (5′–3′) a
pET-28a-*hcp1*-F	GGATCCATGCTGGCCGGAATATATCTCAA
pET-28a-*hcp1*-R	AAGCTTTCAGCCATTCGTCCAGTTTGCGGC
pMLS7-*hcp1*-F	GCTCTAGAATGCTGGCCGGAATATATCTCAAGGTCAAAGG
pMLS7-*hcp1*-R	CCAAGCTTTCAGCCATTCGTCCAGTTTGCGGC
*hcp1*-up-F	TGTAAAACGACGGCCAAGTGCCAAGCTTGTGACCGATCTGCCGCTCTAC
*hcp1*-up-R	CCCGCGACGATTCGCGATCAGCCATTCTGAGATATATTCCGGCCAGCATG
*hcp1*-down-F	GCGCCATGCTGGCCGGAATATATCTCAGAATGGCTGATCGCGAATCGTCG
*hcp1*-down-R	AAACAGCTATGACATGATTACGAATTCAGGCGAACGAGCTCGTCCTCG
pGEX-6P-*hcp1*-F1	CGGGATCCATGCTGGCCGGAATATATCTCAAG
pGEX-6P-*hcp1*-F2	CGGGATCCCGCGGCACGATCACGTTGA
pGEX-6P-*hcp1*-F3	CGGGATCCGCGCTCGGCAAGCGCGAG
pGEX-6P-*hcp1*-R1	CGGAATTCTTATCAGCCATTCGTCCAGTTTGCG
pGEX-6P-*hcp1*-R2	CGGAATTCCAGCACTTTTTCGAACTTGTACGTGAACAG
pGEX-6P-*hcp1*-R3	GGTCTAGATTACGTCTTCGGACGGTGGATCG

a. The underlined base sequence refers to the cleavage site used to clone into the corresponding plasmid by the T4 ligating technique.

## Data Availability

The datasets used and analyzed during the current study are available from the corresponding author on reasonable request.

## Supplementary Materials

The following supporting information can be downloaded at: https://www.mdpi.com/article/10.3390/pathogens13010043/s1, Figure S1: Hcp1 is required for MNGC formation; Figure S2: Preparation of rHcp1; Figure S3: Analysis of anti-rHcp1 mAbs; Figure S4: Subtype and affinity analysis of 13F1; Figure S5: Cartoon (left) and surface (right) of rHcp1 as defined by X-ray structural data (PDB:4TV4).
