# Supplementary material for: Anti-Hcp1 Monoclonal Antibody Is Protective against Burkholderia pseudomallei Infection via Recognizing Amino Acids at Asp95-Leu114
Source: Pathogens. 2023 Dec 31;13(1):43. doi: 10.3390/pathogens13010043 (PMC10818278; doi:10.3390/pathogens13010043)
Supplement: Supplementary file 1 [file pathogens-13-00043-s001.zip › pathogens-2688976-supplementary.pdf]

Supplementary Figure:

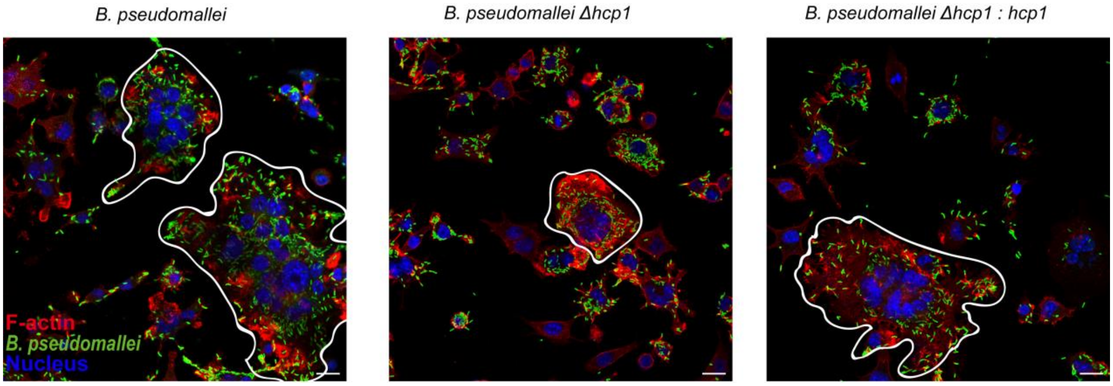

Figure S1. Hcp1 is required for MNGC formation. Bacteria, cytoskeleton, and nucleus were stained with *B. pseudomallei* polyclonal antibodies, Actin-Tracker Red-594, and DAPI. Images were acquired by confocal laser-scanning microscope, and the white line is the MNGC. Scale bar, 20 μm.

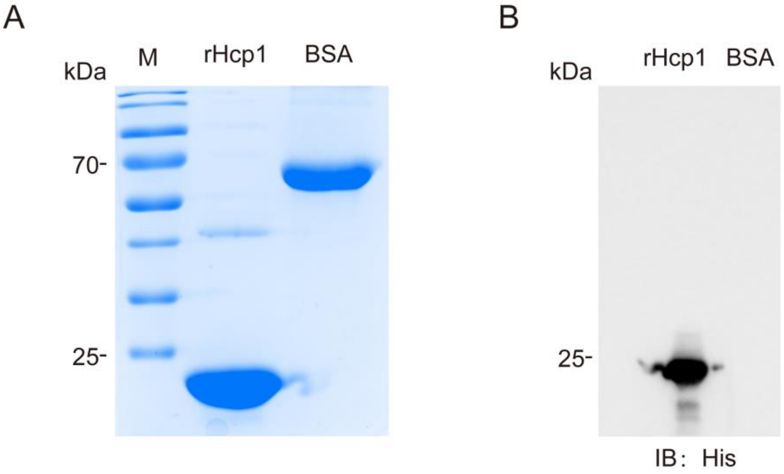

Figure S2. Preparation of rHcp1. (A) The purified rHcp1 was detected by SDS-PAGE. Lane M: protein markers. (B) Western blotting showed that rHcp1 was specifically recognized by anti-His tag antibody.

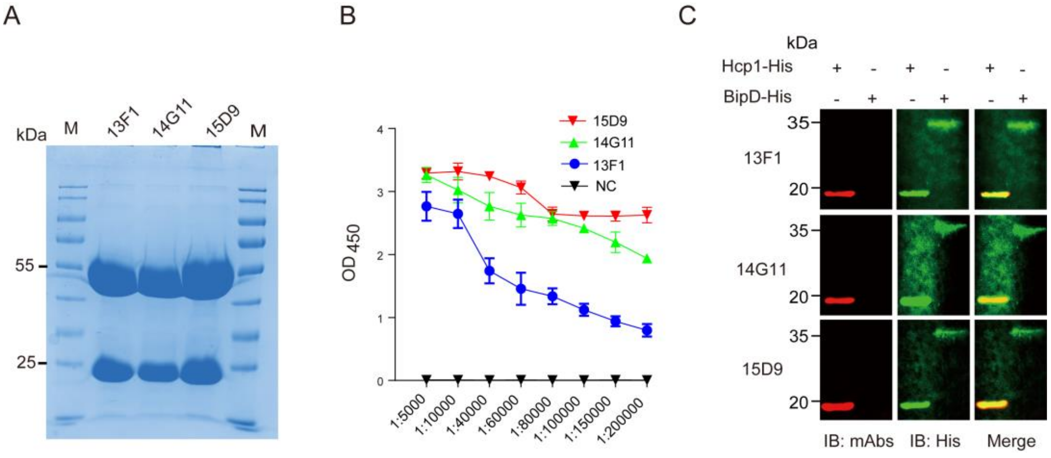

Figure S3. Analysis of anti-rHcp1 mAbs. (A) SDS-PAGE detection of purified anti-rHcp1 mAbs. Lanes 13F1, 14G11, and 15D9: three anti-rHcp1 mAbs; M: protein marker. (B) ELISA measurement of the titer of three mAbs, with the abscissa being the dilution ratio of the mAbs. (C) rHcp1-His and rBipD-His were detected with anti-rHcp1 mAbs and fluorescent secondary antibodies.

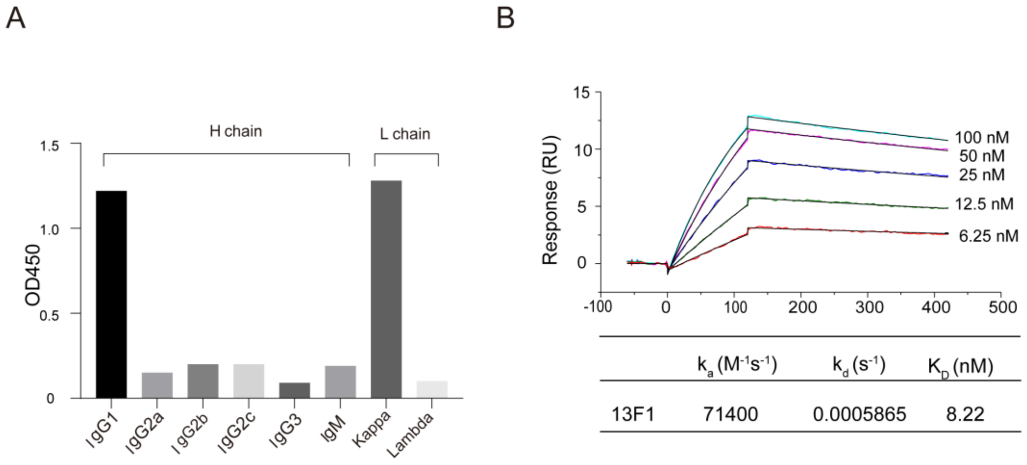

	k_a ($M^{-1}s^{-1}$)	k_d (s^{-1})	K_D (nM)
13F1	71400	0.0005865	8.22

Figure S4. Subtype and affinity analysis of 13F1. (A) Identified the subtype of 13F1 by ELISA. (B) Kinetic interaction of Hcp1 with protective MAb 13F1 as visualized by SPR with a BiacoreT200 machine. The association and dissociation phases were monitored for 120 s by following the change in SPR signal (colored curves) and recording resonance units (RU). Black curves represent the fit of data to a single-site binding model. Kinetic parameters derived from these experiments are listed. The binding phase was used to determine the association constant (ka) between mAbs and Hcp1. The dissociation phase (kd) was measured using the rate of decline in RU on introduction of free buffer at the end of mAbs injections, and the dissociation constant (KD) of the complexes was determined as the ratio kd/ka.

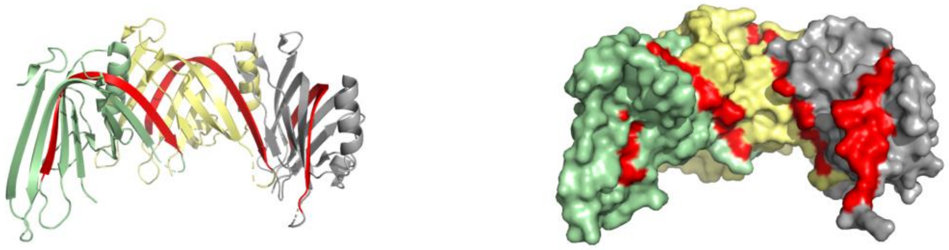

Figure S5. Cartoon (left) and surface (right) of rHcp1 as defined by X-ray structural data (PDB:4TV4) This structure is based on a trimer which consists of three Hcp1 monomers. Amino acid residues in green, yellow and gray represent these three Hcp1 monomers. Amino acids in red (Asp95-Leu114) represent a major epitope recognized by 13F1. Image was created with the PyMOLTM Software.
